# Evaluation of potential antimicrobial chlorhexidine digluconate microencapsulated in model experimental dental biofilm

**DOI:** 10.1186/1753-6561-8-S4-P86

**Published:** 2014-10-01

**Authors:** Giselle Medeiros da Costa One, Allan de Jesus dos Reis Albuquerque, Daniela Correia Cavalcante Souza, Fabio Correia Sampaio

**Affiliations:** 1 Federal University of Paraiba, João Pessoa, Brazil

## Background

The formation of biofilm on the tooth surface is a result of the process of colonization of cariogenic bacteria, especially *Streptococcus mutans*, the main responsible for cariogenic (acid production) of dental biofilms [1]. The removal and chemical control of dental biofilm with chlorhexidine digluconate 0.12% (CLX) has been the most effective antimicrobial agent of choice clinic. However, despite its indisputable efficacy, no side effects such as darkening of the teeth, tongue and restorations, burning mouth and changes in taste. The formation of inclusion compounds CLX for the controlled release of the compound in a lower concentration could be a viable strategy to reduce these side effects without diminishing the therapeutic efficacy of the product [2,3]. Against this background, this study aimed to prepare nanocapsules CLX (processing aid) (1.2%) in model spray drying using modified starch as the encapsulating agent, tested in biofilm model using bovine teeth.

## Methods

The microbiological evaluation of in vitro antimicrobial activity of different formulations was made against *S. mutans *strain UA159 grown in artificial saliva, a model of biofilm produced in the flow cell. The biofilm on the teeth was exposed to nano-CLX, CLX Periogard ^® ^and standard laboratory for 2 minutes. Cell viability was measured by fluorescence LiveDeadBactLightTM Kit with dyes Syto ^® ^9 and Propidium Iodide.

## Results

The results showed equivalence between efficiency and CLX laboratory Periogard ^®^, featuring both 0% percentage of S. mutans after treatment. The CLX nanocoated (1.2%) showed significant efficiency at 90% cell death indicating controlled release of the drug. The model CLX nanocoated by spray drying, and modified starch as encapsulating agents when tested in a model of biofilm using bovine teeth showed good response when used as antimicrobial.

## Conclusions

The molecular nature of chlorhexidine ensures its effectiveness in combating bacterial biofilms, and such activity is in fact already enshrined. However, the alternative formulation nanocapsulada allows an effective control of its release, ensuring control of the main process cariogenic bacteria, thereby reducing their undesirable aesthetic point of view, representing new alternative for the application and release of a single antimicrobial without the immediate need for new drugs.
